# Trilateration of blast wave arrival time: an inverse method for determining explosive yield and position

**DOI:** 10.1098/rsta.2024.0040

**Published:** 2025-09-25

**Authors:** Jay Karlsen, Dain G. Farrimond, Tommy J. Lodge, Samuel E. Rigby, Andrew Tyas, Sam D. Clarke, Timothy R. Brewer

**Affiliations:** ^1^ School of Mechanical, Aerospace and Civil Engineering, The University of Sheffield, Sheffield, UK; ^2^ The Innovation Centre, Blastech LTD, Sheffield, UK; ^3^ Arup Resilience, Security & Risk, Manchester, UK; ^4^ Synthetik Applied Technologies LLC, Austin, TX, USA

**Keywords:** Beirut, blast, explosion, arrival time, inverse method, Monte Carlo

## Abstract

This paper details the development of a rapid inverse approach to determine the yield and location of an explosion through trilateration of empirical laws for blast wave arrival time. A rigorous sensitivity analysis of measurement uncertainty is first performed. From this, a probabilistic framework is proposed that utilizes Monte Carlo sampling of datasets to mitigate the effects of the variability and uncertainties typically present in blast events. Subsequently, the trilateration method is successfully applied to two existing datasets. Analysing well-controlled small-scale laboratory experiments, charge mass is predicted within 6.3% of the true yield, and position within 3.65 charge radii of the true centre. Social media footage of the 2020 Beirut explosion is then used to assess performance against data collected under in-field conditions. The predicted yield of 0.52 kt_${,}_{\text{TNT}}$_ shows good agreement with the literature, and charge position is predicted to within the radius of the crater. Trilateration is shown to be able to rapidly and reliably determine explosive yield and centre, despite large levels of sensor noise. The sub-second computation time of this approach offers the possibility to better model and predict the damage and injury patterns immediately after an explosion, facilitating more effective disaster response planning.

This article is part of the theme issue ‘Frontiers of applied inverse problems in science and engineering’.

## 1. Introduction

Explosive incidents, be they accidental or intentional, can cause considerable damage to their surroundings. After detonation, high pressure and temperature explosive products rapidly expand, displacing and compressing the surrounding air which ‘shocks up’ and propagates at supersonic speed away from the source as a blast wave [1]. Upon impact with obstacles, the blast wave imparts high-magnitude pressures and impulses that have the potential to result in significant injury, life-long disability and death, alongside irreversible structural damage or catastrophic collapse [2].

Following such an event, it is imperative that life-saving action is immediately taken and in the longer term, effective recovery strategies must be applied. A vital contributor to the efficacy of these responses is whether the explosion’s properties are accurately estimated since these can be used to predict the severity and distribution of injury [3] and structural damage [4,5], which aids first responders [6] and assessors of building health [7]. This information can also help to detect any deficiencies in safety protocols that may have led to the event, guiding policy changes [8]. Additionally, a more comprehensive understanding of explosions and their consequences will lead to more accurate quantification of explosive risk [9].

The information available in the immediate aftermath of an explosion relate almost exclusively to human injury, structural damage [10,11] or direct measurements such as seismo-acoustic signals [12–14]. Inverse analysis is made more complex in the case of explosions as the properties of the blast wave are influenced by a broad range of factors, such as the presence of environmental obstacles [15], as well as the properties of the charge itself, including its shape [16], constitution [17], confinement [18] and whether it was detonated in the air, on the surface or underground [19].

Inverse methods incorporating a complete description of such intricacies would be prohibitively time consuming. Instead, analysis of above-ground explosion scenarios may be expedited by assuming the blast behaves similarly to well-known empirical laws. Therefore, quantifying the equivalent yield and centre of an explosion are of paramount importance, since the former effectively captures the magnitude of the blast, while the latter controls the distribution of its effects. Of the existing inverse approaches detailed in the following section, none are capable of estimating both quantities with the accuracy and timeliness required to effectively inform immediate post-blast response efforts. Consequently, this paper develops an inverse technique novel in the field of blast: trilateration. Following the literature review, a conceptual overview of the proposed solution scheme is introduced and its efficacy is assessed against data from real-life explosion case studies. The trilateration method developed herein is shown to display high accuracy, and therefore its effectiveness for use in inverse analysis of blast events is proven.

## 2. Literature review

A number of existing inverse schemes, e.g. [20–22], use the dimensions of the crater formed by a blast to estimate yield. As an extension, Verolme *et al.* [23] apply ballistics theory to inversely estimate equivalent charge size from debris ejected from the crater. As acknowledged by the authors, this requires extensive data collection of parameters such as launch height and angle of debris, lateral travel distance, knowledge of whether the debris ricocheted, in addition to the crater’s location. Consequently, although the authors successfully applied the approach to two case studies, the data gathering process could be prohibitive and limits the wider applicability of debris-throw analysis.

Of the inverse analyses that do not explicitly make use of the crater, many still require knowledge of the position of the charge centre. For example, Jarett [24] proposed a correlation between the distribution of structural damage around the origin of an explosion and its yield, defining regions of similar structural damage by radii from the blast seat and using their relative proportions to estimate charge mass. This work was expanded upon by Gilbert *et al.* [25], seeing successful practical application by van der Voort *et al.* [11] in their inverse analysis of the Enschede firework disaster. The same paper showed an adaptation of this damage distribution framework to be compatible with window breakage in their case study of the 1996 Khobar Towers attack.

Despite these practical successes, Jarett’s [24] equation is only directly applicable with masonry structures and thus has been supplemented by pressure-impulse-damage isocurves that capture the response of different structural systems and materials to blast loading [26]. With these advanced correlations in place, accurate predictions of structural damage can be made for a charge of known size, which provides investigators with an alternative inverse technique. For this, they assume the yield of the explosion for a known or assumed charge centre, simulate the event and compare the computed damage outputs with the real-life effects. By iterating through various masses until alignment is maximized, the true charge yield can be estimated. This simulation tends to be performed with computational fluid dynamics (CFD) software, which utilizes stepwise solutions to the conservation equations to model fluid flow, even in complex environments [27]. Christensen & Hjort [28] successfully applied this process, accurately predicting the yield of the 2012 Oslo bombing from the distribution of glazing damage. A similar methodology is displayed in Ambrosini *et al*.’s [10] inverse investigation into the 1994 Israel–Argentina Mutual Association attack based on structural damage. In this case, the true charge centre was not known exactly, instead the authors assumed it to be somewhere within a 10 m^2^ area (as identified from photographs of the aftermath) before proceeding with their investigation. The introduction of this second unknown required iteration through both mass and position, and so four different assumed yields were simulated at seven strategic locations. From this, the authors reduced the uncertainty in their charge mass estimate by 67%, from a range of 300 to 100 kg_${\;}_{\text{TNT}}$_, and identified the charge position to be within a 1.25 m^2^ area. It must be emphasized that the level of accuracy achieved would have been significantly reduced without the addition of more simulations had the initial 10 m^2^ search area been larger.

CFD is currently the only reliable and accurate tool for modelling blast wave propagation in complex environments [29,30]. This approach, however, requires significant computational time [31], meaning that inverse solvers which utilize high-fidelity CFD simulations are currently ineffective in supporting decision-making immediately after an explosive event. Consequently, it is essential to use simpler, more time-efficient analyses when the circumstances dictate. The simplest scenarios are ‘free-air’ and ‘free-field’ wherein blast wave propagation is wholly unimpeded by obstacles aside from the ground surface in the case of the latter [1]. In these conditions, the semi-empirical predictions of Kingery & Bulmash (KB) [32] are regarded as accurate estimators of far-field blast wave parameters for a large range of scaled distances [33–35]. Rigby *et al.* [36] incorporated this predictive tool into their analysis of the 2020 Beirut explosion, assuming a charge seat and iterating through values of assumed mass until the residual error between measured and predicted blast wave time of arrival was minimized. Despite occurring in an urban area, differences between the true blast propagation behaviour and the assumption of free-field conditions in the inverse model were deemed negligible because the physical scale of the blast wave was considerably larger than the buildings it encountered. More typical city-based explosions—including intentional attacks with even the largest improvised explosive devices [37]—are likely to have a yield hundreds or thousands of times lower than the Beirut explosion, thus environmental geometry will likely play a significant role in blast wave propagation.

In their comprehensive review of the state of post-blast assessment, Shi *et al.* [38] stated that this field of study ‘*remains in its infancy*’, citing a lack of overall robustness and efficiency, and the necessity for advancements in CFD. This itself is contingent upon improvement in experimentation to ensure rigorous validation [15]. One factor contributing to the lack of robustness of existing inverse approaches is the fact that the location of the charge centre must be known in advance for many of the established methodologies to function, including all of those previously discussed. In some practical cases, however, this information may be unavailable, i.e. low height of burst scenarios that may result in minimal cratering, or craters being back-filled by rubble from collapsed structures.

This limitation has been somewhat addressed by a selection of inverse schemes capable of determining charge centre. One such method is proposed by Li *et al.* [39], who employed image interpretation on two sources of video footage of the 2015 Tianjin explosion in order to approximate the origin of the blast. By enhancing the quality of the images using varying processing techniques, the authors obtained sufficient evidence to ‘*significantly narrow down the list of potential blast seats*’. While this approach clearly has potential in locating the charge centre, it is unable to do so definitively, as stated by the authors themselves. Additionally, the image interpretation presented by Li *et al.* [39] provides no scope for yield estimation.

An alternative approach which captures estimates of both charge centre and yield utilizes seismo-acoustic data [38], e.g. the work of Ceranna *et al.* [40] in response to the 2005 Buncefield explosion where the decay in recorded air pressure with distance was used to estimate TNT equivalent charge mass, and back-azimuth inversion of seismograph data enabled an estimate of charge centre. In this case, the authors estimated yield to the correct order of magnitude, but the error in position was 35 km. Using a similar approach, Evers *et al.* [41] estimated the location of the 2004 Ghislenghien gas-pipe explosion with a radial error of 13 km. The yield of these events was of the order of magnitude of tens of thousands of kilograms of TNT equivalent, while more typical urban explosions—particularly intentional ones—have a yield closer to hundreds of kilograms of TNT equivalent [37]. As the yield of an event reduces, the position estimation accuracy decreases due to a greater amount of relative noise in recorded data and because seismic waves decay before being detected by sensor arrays [42]. Therefore it is unlikely that the analysis of smaller-scale scenarios using this approach will result in practically useful position outputs.

Hou *et al.* [43] developed an inverse approach to approximate charge position (for a given yield) by using the UFC-03-340-02 [44] relations to back-calculate scaled distance from overpressure. When applied to free-air experimental data, the authors achieved a position estimate with an absolute radial error of 84 mm, although the relative scale of this error is unknown as charge yield was not specified. The authors then applied the same technique, with an alternative overpressure relationship derived from numerical modelling [45], to a scenario with an explosive moving with a vertical velocity prior to detonation. The radial error in estimated position increased to 1.68 m. In its current form, however, the method requires manual removal of noisy measurements and the presence of variability in just two of the measurements induced a 50% increase in position error, therefore it has a low robustness and high sensitivity to typical sources of variation seen in real-world data. This is particularly true for peak reflected overpressure, which is known to exhibit considerably higher variability than time of arrival [46,47].

In summary, there is the need for an efficient inverse tool capable of accurately estimating both the yield and the centre of an equivalent charge, in order to facilitate more effective response efforts and decision-making. However, at present, existing methods are either time-prohibitive or unable to accurately estimate both yield and position. To address this, the current paper proposes and evaluates the use of trilateration as an inverse analytical methodology for use in blast applications.

## 3. Development of trilateration for post-blast inverse analysis

### (a) Foundational principles

**Figure 1.**
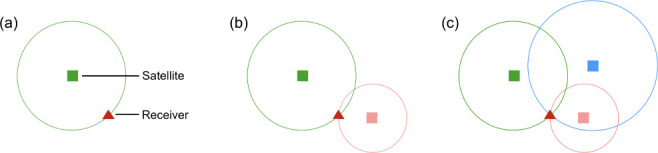
Illustration of two-dimensional trilateration in the context of GPS. (*a*) One satellite, infinite potential solutions. (*b*) Two satellites, two potential solutions. (*c*) Three satellites, a single valid solution.

The inverse scheme in this paper is based on ‘trilateration’, which is otherwise deployed for location sourcing in various applications from seismology [48] to the Global Positioning System (GPS) [49]. In the latter, trilateration is used to estimate the geospatial coordinates of a receiver by measuring its distance from a number of satellites at known locations. This is shown schematically in figure 1 (in two dimensions) whereby the location of the receiver is given by the intersection between overlapping circles (termed ‘solution circles’), each of which denote the possible locations of the receiver around a given satellite.

In a free-field blast setting with a hemispherical charge, the properties of a blast wave are governed exclusively by the equivalent yield of the explosive, W, and the distance from the charge’s centre to the measurement location, often termed ‘stand-off’, R, i.e. f(R,W) [32]. For a hypothetical inverse blast analysis where the value of the true charge yield, $W_{true}$, is known, the true stand-off distance between the explosive and the gauge location can therefore be inversely computed directly from the blast wave property values measured at said gauge. In so doing, a solution circle of radius R centred on the gauge manifests, representing the reduced set of circumferential locations where the charge could be positioned. Similar to figure 1*c*, by repeating this procedure with sufficient gauges, the set of potential charge locations reduces to a single point that is identified by the mutual intersection of each gauge’s solution circle. The conceptual link between GPS positioning and free-field blast problems is clear, albeit in the former the inverse problem is solved with multiple sources and a single receiver, whereas the latter comprises a single source and multiple receivers.

The above procedure necessitates a reliable relationship between charge yield, stand-off and some measured blast effect. For this purpose, equation (3.1)^1^ is adopted throughout and relates the measured relative blast wave time of arrival, $t_{a}$, to R and W (as defined previously). Although time of arrival (TOA) is adopted as the measured blast effect throughout this work, the methodology itself can operate with any other quantity that is also a function of yield and stand-off (e.g. structural damage, human injury or other blast parameters such as pressure and impulse, etc.). Similarly, any empirical, numerical or analytical blast parameter predictor may be used in place of equation (3.1), provided it is compatible with the problem’s measured effect data.


(3.1) $$\begin{array}{rl} \log\left(\frac{t_{a}}{W^{\frac{1}{3}}}\right) & =0.0717\cdot {\left(\log\frac{R}{W^{\frac{1}{3}}}\right)}^{5}-0.0567\cdot {\left(\log\frac{R}{W^{\frac{1}{3}}}\right)}^{4}-0.3192\cdot {\left(\log\frac{R}{W^{\frac{1}{3}}}\right)}^{3}\\ & +0.1495\cdot {\left(\log\frac{R}{W^{\frac{1}{3}}}\right)}^{2}+1.8165\cdot \log\frac{R}{W^{\frac{1}{3}}}-0.3215 \end{array}$$

In practical blast scenarios, however, the value of $W_{true}$ is likely to be unknown, thus making it a target output of the inversion. The subsequent sections thus develop and validate a generalization of the above, integrating charge yield as an unknown to be found alongside the true charge position.

Note, the blast scenarios considered throughout the remainder of this work involve surface charges with source and sensor locations all located at (approximately) ground level, reducing the inverse problems to two dimensions. Generalizing the algorithm from two to three dimensions requires only minor changes to the methodology described in this paper. Initially, test data are generated directly from equation (3.1), and subsequently the trilateration scheme is trialled on real-life experimental data with varying levels of noise and uncertainty.

### (b) Outlining the inverse algorithm

First, it is assumed that all sensors are synchronized and recording with a common time base. Absolute time of arrival can be converted to the necessary relative TOA by setting detonation to be time-zero. The implications of detonation time being unknown are explored later in this article.

As previously described, the point of mutual intersection between the solution circles of all gauges can only arise when the value for W used in the calculation of the gauge stand-off distances exactly equals $W_{true}$. Therefore, when $W_{true}$ is unknown, the trilateration procedure can be repeated iteratively with different assumed values of W until the mutual intersection point manifests. As before, the coordinates of this mutual intersection are those of the true charge centre, and the value of W that generated it must be exactly equal to the true charge yield.

‘Exhaustive search’ is the algorithm adopted for the iterative process. It increments the assumed value for W, by a user-defined amount, w, ensuring that the solution domain is rigorously searched with the desired precision. The range of assumed values of W to be considered must be bounded by some extremes, $W_{min}$ and $W_{max}$, that are also to be specified when the inversion is initialized.

**Figure 2.**
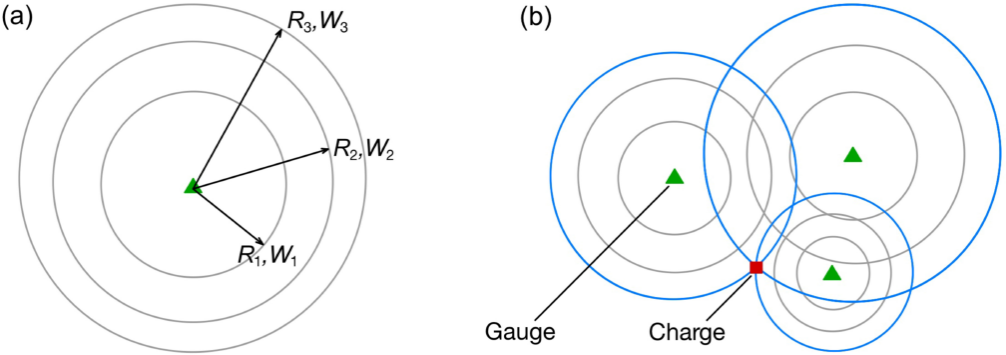
Illustration of the concentric solution circles associated with the incrementation of assumed yield. (*a*) Several potential stand-off distances calculated for different assumed values of yield. (*b*) Trilateration when both charge yield and position are unknown. The highlighted solution circles produce a mutual intersection at the true charge position; the value of assumed yield used must be exactly equal to the true charge yield.

Throughout the above, equation (3.1) is again used to calculate the scaled distance, $R/W^{1/3}$, between gauge and charge using the TOA measured at said gauge. Where before this could be converted into a single value for stand-off, with $W_{true}$ unknown, various values of W are assumed and trialled in its place. This generates many potential charge stand-off distances, each paired with a different assumed yield, all of which are valid potential solutions in accordance with Hopkinson–Cranz scaling^2^ [50,51]. This can be visualized in figure 2*a* as a series of concentric solution circles centred on the gauge, where each circle radius (stand-off, R) is associated with a different assumed value of W. Figure 2*b* depicts how, theoretically, continued iteration will identify a value of W that induces a point of mutual intersection among all gauges.

To ascertain the presence and location of the point of mutual intersection for a given assumed value of W, the algorithm serially calculates all points of intersection between each unique pair of solution circles within the set of gauges. For any two solution circles centred on (a,b) and (c,d), respectively, with stand-off radii $R_{1}$ and $R_{2}$, the coordinates are calculated by solving equations (3.2)—(3.3) simultaneously.


(3.2) $$\begin{array}{lcr} & (x-a{)}^{2}+(y-b{)}^{2}=R_{1}^{2} & \end{array}$$

(3.3) $$\begin{array}{lcr} & (x-c{)}^{2}+(y-d{)}^{2}=R_{2}^{2} & \end{array}$$

Solution of the simultaneous equations generates equations (3.4)–(3.5), where r is the distance between circle centres as per equation (3.6). These are used to compute the intersection coordinates, x and y, directly. Should all solution circle pairs output a common point of coincidence, then the mutual intersection is identified.


(3.4) $$x=\frac{1}{2}(a+c)+\frac{R_{1}^{2}-R_{2}^{2}}{2r^{2}}\cdot (c-a)\pm \frac{1}{2}(d-b)\cdot \sqrt{2\cdot \frac{R_{1}^{2}+R_{2}^{2}}{r^{2}}-\frac{(R_{1}^{2}-R_{2}^{2}{)}^{2}}{r^{4}}-1}$$

(3.5) $$y=\frac{1}{2}(b+d)+\frac{R_{1}^{2}-R_{2}^{2}}{2r^{2}}\cdot (d-b)\pm \frac{1}{2}(c-a)\cdot \sqrt{2\cdot \frac{R_{1}^{2}+R_{2}^{2}}{r^{2}}-\frac{(R_{1}^{2}-R_{2}^{2}{)}^{2}}{r^{4}}-1},$$

where


(3.6) $$r=\sqrt{(c-a{)}^{2}+(d-b{)}^{2}}.$$

However, the manifestation of this mutual intersection is extremely unlikely, as the introduction of even a small amount of measurement variability would entirely prevent the necessary convergence. Additionally, to support a search sufficiently precise to trial the exact value of the true charge yield, a high-fidelity mass increment must be adopted that would impractically escalate computational expense despite the quick-running nature of the polylogarithmic effect predictor adopted. Therefore, it is necessary that the algorithm be revised, facilitating a suitably accurate estimation of the true charge yield and position should the mutual intersection not arise across the iterations. This is developed subsequently through the proposition of a new convergence metric and the finalized inversion scheme is then validated using a series of well-posed problems ahead of its practical application.

### (c) Integration of robustness

**Figure 3.**
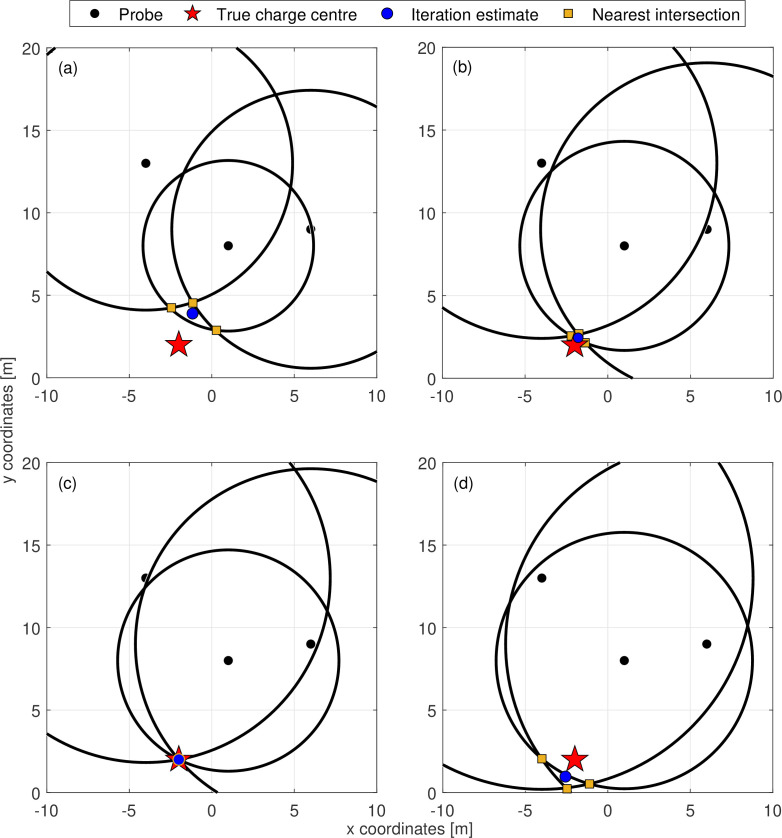
Evolution of the solver across multiple iterations. (*a*) $W<W_{true}$, (*b*) $W\approx W_{true}$, (*c*) $W=W_{true}$, (*d*) $W>W_{true}$.

Figure 3 shows an example of the trilateration inverse scheme, with three pressure gauges in a 20 × 20 m domain. The values assumed for W range between 1 and 150 kg_${,}_{\text{TNT}}$_^3^. The true charge centre is depicted and the true charge yield, $W_{true}$, is 30 kg_${,}_{\text{TNT}}$_. As the assumed yield approaches 30 kg_${,}_{\text{TNT}}$_, the solution circle intersections that are highlighted begin to converge on the true charge centre—becoming more concentrated—then, once the mass iteration exceeds the true value, they diverge again. Therefore, a measurement of the concentration of these intersection points is indicative of the quality of each iteration’s estimate of true charge yield. This value is termed ‘internal error’, ε, and is defined in equation (3.7) as the average distance of n circle intersections from their mutual centroid, (x,y). The centroid of the intersection points is calculated via equation (3.8).

The goal of the iterative scheme is therefore to identify the value of assumed yield for which ε is minimal, as this represents the conditions closest to forming the mutual intersection and thus is indicative of an inverse estimate that best represents the true event’s initial conditions.


(3.7) ε=1n∑i=1n(x−xi)2+(y−yi)2

(3.8) (x,y)=(1n∑i=1nxi,1n∑i=1nyi)

The centroid of the intersection points is taken as the iteration’s estimate of charge centre. As per figure 3*c*, it aligns exactly with the true position when the true charge yield is input (and the TOA measurements are noiseless). Luo *et al.* [52] use this same principle when working to improve the robustness of trilateration navigation systems under noisy conditions.

As described previously, the intersection coordinates of any two circles can be found using equations (3.2)–(3.6). The number of real roots to these equations depends on whether the circles intersect once, twice, infinitely or not at all. An infinite number of intersections can immediately be neglected from the analysis as this would imply two probes occupying the same space, which is non-physical. As a safeguard, the algorithm includes a simple logic gate that ensures the origin coordinates of the two circles of interest, from equations (3.2) and (3.3), are distinct. The other three solutions are all plausible, and their implications for the algorithmic procedure are as follows:

(a) A single intersection is trivial.
(b) Should a pair of circles intersect twice then the algorithm must be able to identify which of the two is more likely to be correct (given that there is only one charge and thus only one true centre). This principle is clear in figure 3 where only half of the intersections converge on the true charge centre. Thus, the algorithm is designed to determine which of the intersections of any pair of circles is closest to other intersection points, since a more concentrated group of intersections is more likely to form near the true solution.
(c) When two circles do not intersect, the solutions to their simultaneous equations are imaginary. Given that this is non-physical, the scheme must be robust enough to discount them. Fewer intersections means that when the internal error of the final group is measured, the apparent error may be artificially low due to there being fewer points included in the calculation. In response, a provision has been made such that the internal error value will augment by a large real number, K, for each of the j number instances wherein two circles do not intersect. K is defined herein as being equal to the stand-off distance of the probe recording the maximum measured TOA, calculated with the upper limit of assumed yield, $W_{max}$. Accordingly, equation (3.8) is updated to equation (3.9).
(3.9) ε=1n(j⋅K+∑i=1n(x−xi)2+(y−yi)2)
  The factor K also ensures that iterations with fewer missing intersections are considered to be better quality than those with a greater number of missing intersections.

**Figure 4.**
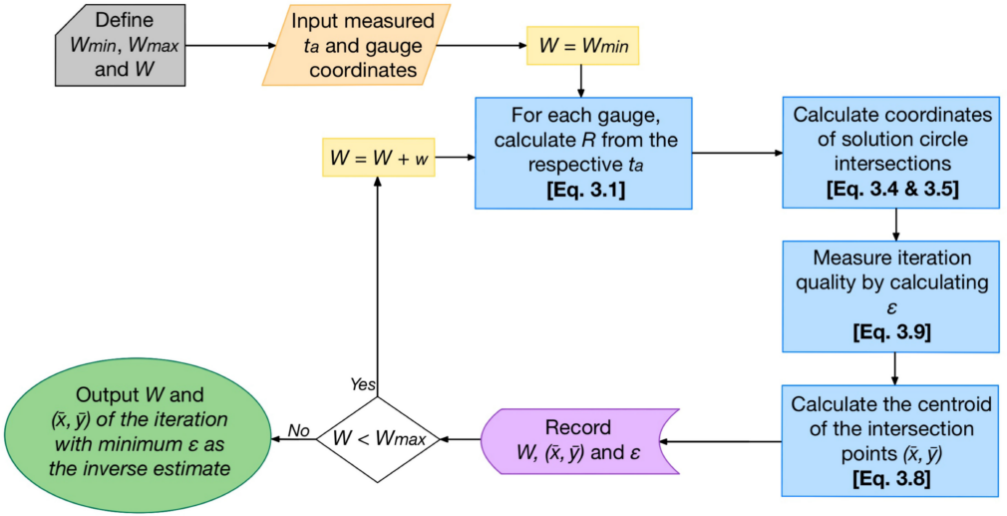
A flowchart describing the initialization and algorithmic procedure for the developed post-blast trilateration routine.

Following the incorporation of the revised internal error function, figure 4 summarizes the finalized trilateration algorithm. As described, the exhaustive search scheme is initialized by the user defining the range of assumed charge yield values to be trialled (bounded by the upper and lower limits $W_{min}$ and $W_{max}$), as well as the fidelity of the searching increment, w.

### (d) Applying and testing the algorithm

**Figure 5.**
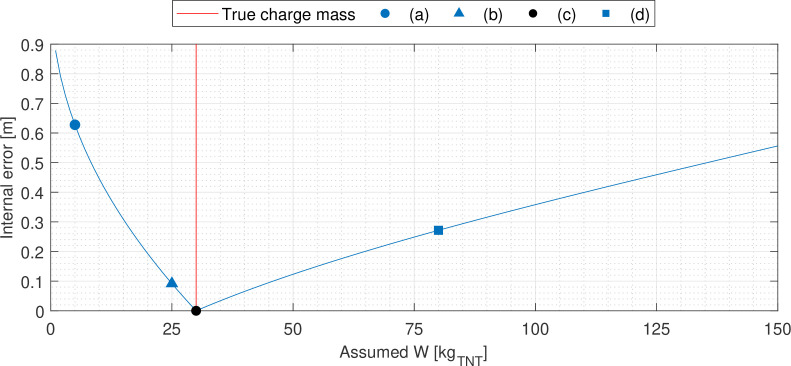
Representative internal error profile of the trilateration scheme. The internal error values of iterations extracted in figure 3a-d are annotated.

To ensure functionality, the trilateration algorithm is first applied to the inverse problem described in the previous section. Figure 5 depicts the corresponding internal error profile, with markers denoting the errors of each of the iterations in figure 3. It is apparent that the internal error successfully approaches zero as the assumed TNT equivalent mass approaches the true value, becoming exactly zero when the input value exactly equals the true yield. The assumed value for W that produced this minimum error is identified as the scheme’s best estimate for the true charge yield, and the intersection centroid for this iteration is then used to return an estimate of the true charge location. These inverse estimations are equal to the true initial conditions, verifying that the algorithm functions as intended. Additionally due to the quick-running nature of equation (3.1), the scheme can continue to be solved using exhaustive search without any need for more sophisticated root-finding approaches; this example was completed in just 0.08 seconds for a step size of 0.5 kg_${,}_{\text{TNT}}$_. Therefore trilateration appears to be an effective and efficient technique for the purposes of post-blast analysis when the detonation time is known and the data are without noise. Both of these idealizations will be addressed subsequently.

Note, this test was conducted such that the charge yield step size enabled one of the assumed masses to exactly equal the true value during testing. Coarser yield step sizes were also trialled, and it was found that the minimum error always occurred at the trialled mass closest to the true solution. This suggests that, in its simplest form, the inverse trilateration approach with an exhaustive search algorithm is sufficient to approximately determine the solution to any desired half-step accuracy, thereby informing a more refined, sophisticated search over a reduced domain, as discussed in §2. A decrease in step size results in an approximately linear increase in computational time.

### (e) Updating the scheme for an unknown detonation time

In some cases, the time of detonation may be unknown, and therefore the relative time of arrival for each probe cannot be determined since absolute detonation time is an additional variable. To incorporate this, the existing algorithm can be adapted to iterate through both assumed yield *and* assumed detonation time, thereby permitting relative TOA values to be calculated for each sub-iteration. For brevity, the results of this have been excluded as ultimate performance is very similar to the known TOA scheme provided the detonation time increment is sufficiently small, however, there are substantial increases in computation time and storage requirements.

## 4. Evaluating performance when input data contain noise

### (a) Common sources of noise

Thus far, the algorithm has been tested exclusively on noiseless data obtained directly from equation (3.1). Consequently, it is important to establish the sensitivity of the trilateration inverse scheme to measurement variability.

With respect to blast scenarios, example sources of measurement error include:

(i) *Sample rate rounding*. The gauges used to measure blast parameter values, in this case TOA, take readings at specific intervals defined by their sampling rate [53]. Should the blast wave arrive between samples, then the recorded arrival time will be rounded up to the next sample, inducing an error proportional to the sample rate of the gauge. Rigby *et al.* [36] demonstrated that video footage of a blast recorded on smartphones can be a useful method of data collection and the typical recording equipment involved, microphones and smartphone video cameras, sample at 44 100 Hz [54] and approximately 30 Hz [55], respectively. Consequently, the maximum error in TOA imparted by these will be 0.023 and 33.3 ms, respectively.
(ii) *Blast wave inhomogeneity*. It is known that the initial propagation of the blast wave is highly non-uniform as it is driven by the chaotic expansion of detonation product and affected by internal pressure gradients [56]. This non-uniformity gradually becomes negligible as the blast wave enters the far-field [47], but it is prevalent in the near-field. Both the magnitude and variability of the imparted noise, and thus the resulting deviation from the KB relations, are unquantifiable at present as there is a dearth of reliable near-field data, given the extreme conditions and complexity of measurement [57]. Consequently, blast behaviour in this range is poorly understood [58].
(iii) *Epistemic uncertainty*. Any deviation of the true blast radius–time profile from the KB idealization due to external factors such as charge shape, presence of casing, detonator location, atmospheric effects, etc., which are not incorporated in the model.

Robustness of the trilateration inverse scheme to varying levels of input variability is assessed in order to reflect the large range of practical sources and types of noise discussed above. Accordingly, a Monte Carlo framework is adopted hereafter.

### (b) Output accuracy sensitivity assessment

In a Monte Carlo framework, batches of input data are stochastically generated according to some probabilistic function and analysed [59]. The mean output of all the batches is representative of typical performance of the analysis for the assigned input variability. In this case, TOA data are generated with an imposed noise randomly selected from a normally distributed error profile. The profile has a mean of zero per cent relative error and a user-defined standard deviation, allowing for adjustments to the severity of noise to model different sources of error as discussed previously. For each assigned standard deviation, 10 000 batches of TOA data are generated to evaluate the algorithm’s mean performance for the given variability; this is represented by the mean relative error in estimated yield and charge position.

**Figure 6.**
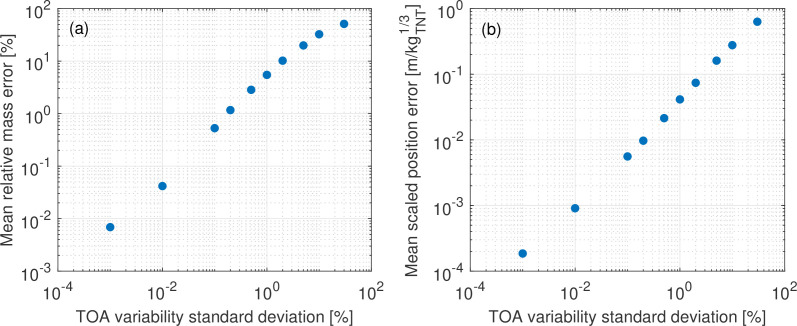
The mean effect of artificial input variability on estimate accuracy. (*a*) Mass estimation. (*b*) Position estimation.

The results of the Monte Carlo analysis are shown in figure 6. As expected, output accuracy decreases when the relative severity of noise increases. This also enables an estimation of confidence in the inverse scheme’s outputs if the statistical profile of a source of noise (e.g. sample rate rounding) is known in advance.

Various laboratory-based free-field experiments have shown TOA measurements to typically lie within 2% of those predicted by the KB relations [34,46,60–62] due to the superposition of various noise sources. When this noise is applied to a typical scenario using four probes (the minimum amount of sensors required for the scheme to perform consistently in two dimensions, with noise and an unknown charge mass), figure 6 shows the trilateration inverse scheme can be expected to estimate equivalent charge yield with an average error below 10.2%, and position with a mean radial error less than 0.08 m/kg${\;}_{{\text{TNT}}^{1/3}}$.

### (c) Optimal quantity of input data

**Figure 7.**
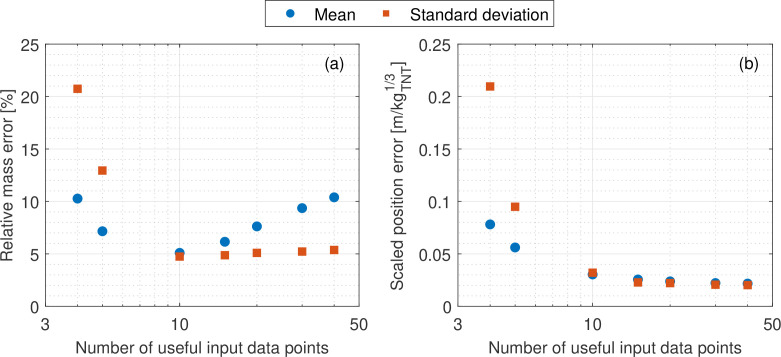
The mean effect of input data quantity on estimate accuracy. (*a*) Mass estimation. (*b*) Position estimation.

Section 4b ascertained the trilateration scheme’s mean performance when analysing a typical scenario using the minimum required amount of input data. Following this, the effect of different quantities of input measurements on the estimate accuracy are evaluated, again in a Monte Carlo framework. The amount of available TOA data was systematically varied, while the standard deviation of noise variability was kept constant at the representative value of 2%. For each total number of sensors, the trilateration inverse scheme was tested on 10 000 distinct scenarios. Each of these had unique combinations of randomly generated charge position, charge mass and gauge locations (again in a 20 × 20 m domain, with $W_{true}$ randomly selected between 1 and 150 kg_${,}_{\text{TNT}}$_ to a ±10 mg precision, and with the assumed W varied by 0.1 kg_${,}_{\text{TNT}}$_ increments). The mean error in estimated mass and position was calculated for each number of sensors, with the results displayed in figure 7.

With respect to position, figure 7*b* shows that accuracy and consistency improves as expected with increased data availability. There are diminishing returns to this effect due to the assumed normally distributed variability profile and so the position error roughly minimizes to a value of 0.025 m/kg${{,}_{\text{TNT}}}^{1/3}$. This accuracy plateau can be achieved most efficiently with a 10 probe analysis; using more gauges increases the computation time for little appreciable improvement in accuracy.

Figure 7*a*, however, demonstrates that the yield estimation behaves unexpectedly. Rather than improving continually with the addition of more input data, there is an apparent minimum estimate error at 10 unique data points before the accuracy again decreases. As more probes are added, the quadratically increasing number of intersections means that there is often as much convergence among subgroups as there is divergence, particularly for iterations of assumed mass slightly above the true value. This is partly because the change in stand-off between iterations is only proportional to the cube-root of the change in mass. Altogether, this prevents the algorithm from differentiating between the performance of different iterations, making the scheme prone to incorrect estimation with larger quantities of noisy data. As such, the optimal quantity of input data is shown to be 10.

In many practical scenarios, however, the quantity of available gauges may be below 10. In these cases, figure 7 can be used to determine indicative confidence intervals on the accuracy of the trilateration scheme. Should more than 10 measurements be available, repeat analyses may be undertaken with subsets of the original data in a Monte Carlo framework in order to reduce the effects of noise. This is demonstrated in §5b with a practical example relating to the 2020 Beirut explosion.

## 5. Experimental validation

### (a) Laboratory-based experiment: PE10 TNT-equivalence

Thus far, trilateration has proven to be an effective inverse tool in the analysis of both ideal and noisy settings. However, until now, the method has only been tested on ‘pure’ data, i.e. that which are generated by the same model that the inverse scheme utilizes. Therefore, it is necessary to assess the trilateration inverse scheme against real-world experimental data.

**Figure 8.**
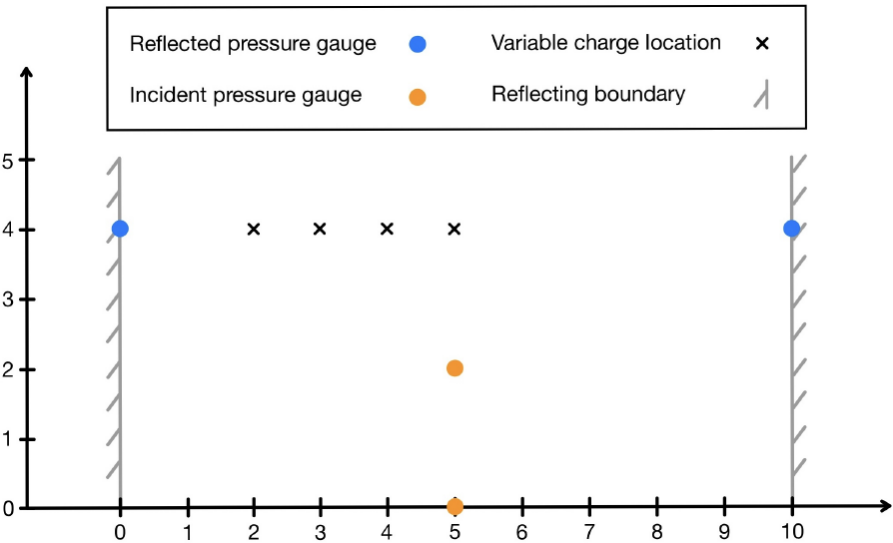
Schematic of the free-field experimental set-up, adapted from Farrimond *et al.* [60]. All dimensions in metres.

Farrimond *et al.* [60] performed free-field experiments with different RDX and PETN-based explosives—namely, PE4, PE8 and PE10—in order to characterize their TNT-equivalence. Blast parameters, including time of arrival, were measured in the set-up shown schematically in figure 8. In each test, the charge was in one of four locations in order to maximize the range of scaled distances for which measurements were available. While only the reflected gauge data were published in the original paper, incident TOA data from the six tests using 250 g${,}_{\text{PE10}}$ hemispheres are utilized here. Since the KB relations require the explosive mass to be input as an equivalent mass of TNT, an equivalence of 1.22 was used, as determined by the aforementioned experimental work.

**Figure 9.**
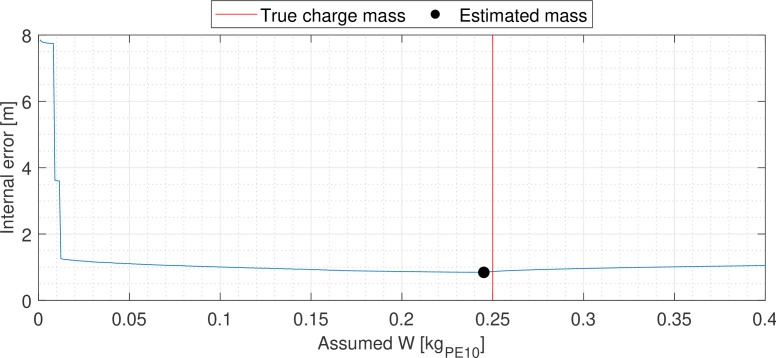
A representative internal error profile during the inverse analysis of PE10 test data.

**Figure 10.**
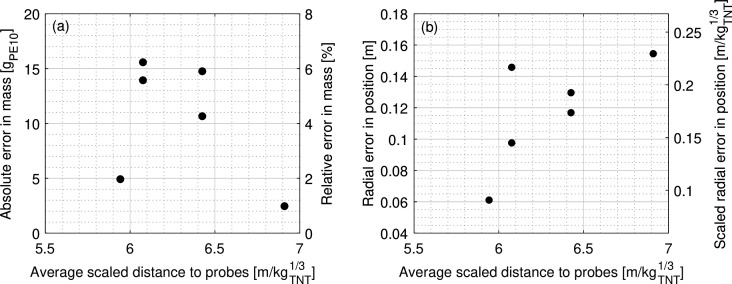
Accuracy of the trilateration algorithm's parameter estimates for the six PE10 experimental datasets. (*a*) Error in the estimated mass. (*b*) Radial error of the estimated charge position.

The trilateration inverse scheme was applied to each of the six experiments, with assumed charge mass varied between 1 g and 1 kg${,}_{\text{PE10}}$ in increments of 1 g. Figure 9 displays an internal error profile from one of these inverse schemes, where the error in estimated mass is 5 g${,}_{\text{PE10}}$, and the corresponding error in position is 90 mm. Figure 10 summarizes the error in mass and position for all six tests.

In all six experiments analysed, the scheme is able to determine charge mass with a mean error of 4.2%, and maximum error not exceeding 6.3%. The algorithm is also able to identify the true charge centre to within 0.12 m on average, which is a scaled distance of 0.18 m/kg${{,}_{\text{TNT}}}^{1/3}$ and no greater than 3.6% of the average distance between the charge and the pressure gauges. Taking the nominal density of the PE10 charge to be the reported 1.55 g/cm^1/3^ [60], the maximum position error does not exceed 3.65 charge radii. This is a clear indication that the trilateration scheme can inversely determine the properties of an explosion from well-controlled laboratory data.

### (b) In-field analysis: the Beirut explosion

#### (i) Context and data availability

Following the successful application of the trilateration algorithm to laboratory-based experimental data in §5a, its performance shall be tested on an in-field scenario with substantial, unquantifiable amounts of measurement noise.

The 2020 explosion in the Port of Beirut is one of the largest in recent history [63]. Up to 2750 tonnes of ammonium nitrate (AN) were reported to have detonated, leading to 15 billion USD in property damage and the deaths of more than 200 people [64]. The scale of this disaster has led to a diverse research response among the blast community, for example, the estimation of injury and structural damage distribution to assist in remediation [64], along with discussion on how best to minimize the likelihood of urban explosions and mitigate their damage going forwards [65].

As part of this research effort, much work has been undertaken to inversely quantify the equivalent TNT yield of the explosion. Rigby *et al.* [36] analysed TOA measurements and concluded 0.5 kt${,}_{\text{TNT}}$ to be most representative of the TNT equivalent charge mass. Stennett *et al.* [66] and Pasman *et al.* [67], utilizing a similar approach but with independent data, found the yield to be within the ranges of 0.407−0.936 and 0.3−0.7 kt_${,}_{\text{TNT}}$_, respectively. Pasman *et al.* [67] also conducted a separate analysis of the cratering, which identified the equivalent yield to be between 0.3 and 1.0 kt_${,}_{\text{TNT}}$_. Using the dimensional analysis of Taylor [68], Díaz [69] concluded the equivalent yield to be within the range of 0.3−0.9 kt_${,}_{\text{TNT}}$_ while, with the same method, Aouad *et al.* [70] calculated it to be 0.235−0.405 kt_${,}_{\text{TNT}}$_. It must be noted that none of these approaches estimate charge position, aside from the crater analysis for which it is implicit, and many must assume it as a prerequisite to their analyses (for which they took the geometric centre of the warehouse storing the detonated AN).

As a result of these numerous independent investigations, the measured parameter data necessary to enable a trilateration analysis of the Beirut explosion are available. Additionally, they provide a wealth of TNT equivalent mass estimations from a variety of established inverse methods, readily facilitating comparison with the proposed algorithm’s yield estimate and thus the evaluation of its performance.

The Beirut explosion occurred inside a dense, complex cityscape, and as such, the propagation of the blast wave cannot ordinarily be considered ‘free-field’. However, other researchers analysing the event have concluded the scale of the explosion to be so great, due to the size of the equivalent charge, that the blast wave’s propagation was largely unimpeded, meaning it can be approximated as meeting trilateration's free-field constraint [36,69]. Note, this approximation will impart additional noise and variability on account of epistemic uncertainty, as discussed previously.

Rigby *et al*.’s [36] TOA data shall be used herein. Said data, presented as supplementary material to the original article, consist of 38 relative arrival time measurements extracted from video footage of the event, alongside the corresponding latitude/longitude coordinates of the approximate camera locations that were determined using a comparison of the footage with Google Street View and open-source mapping data. These polar coordinates are converted to a Cartesian grid, neglecting changes in ground elevation.

To facilitate an evaluation of trilateration’s inverse charge position estimation, the coordinates of the true equivalent charge centre are assumed to be those of the in-plan centroid of the warehouse that contained the AN. Rigby *et al.* [36] also assumed this to be the true equivalent charge position, and Yu *et al*.’s [71] crater analysis shows it to be approximately correct (though Hubbard *et al*.’s [72] rendering implies it may be several metres further north).

#### (ii) Monte Carlo analysis

As discussed in §4c, accuracy of the algorithm is dependent on the quantity of input data: too little and performance is skewed by noise; too much and the method can no longer effectively distinguish iteration quality. Ten input probes were found to be optimal, hence the Beirut explosion shall be evaluated in a Monte Carlo framework that samples the original 38 TOA measurements. Ten thousand simulations were conducted with unique combinations of 10 probes^4^ and an equivalent yield search range of 0.025−2.20 kt_${,}_{\text{TNT}}$_, in 5 t_${,}_{\text{TNT}}$_ increments. Note, to determine the upper bound to this range, the TNT equivalence of the 2750 tonnes of AN was considered. Although pure AN has a TNT equivalence of approximately 0.4 [73], when combined with additives and fuel oil to make ANFO, the equivalence at large scale becomes 0.82 [74] and so this was conservatively assumed as the upper bound for the analysis.

**Figure 11.**
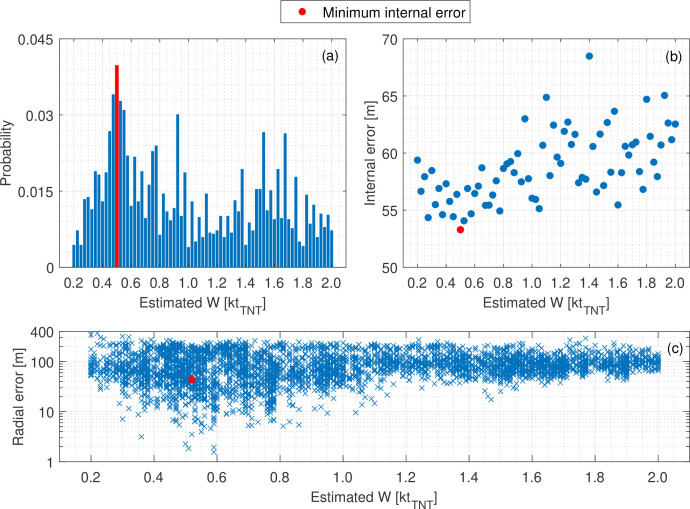
Inverse analysis of the Beirut explosion using trilateration of TOA data. (*a*) A histogram of the estimated equivalent yield outputs with 0.025 kt_${,}_{\text{TNT}}$_ bins. (*b*) Mean internal error of each bin. (*c*) Radial position error of each iteration.

Results from the Monte Carlo analysis are shown in figure 11, with the histograms grouping the yield estimates in bins of size 0.025 kt_${,}_{\text{TNT}}$_. Outputs falling in the upper and lower 10^th^ percentile have been neglected from the probability analysis as their contents are predominantly erroneous. This is because iterations with input combinations with compounding noise artificially gravitate towards the extremes as it is impossible for the algorithm to satisfy their constraints for the assumed masses in the defined range.

**Figure 12.**
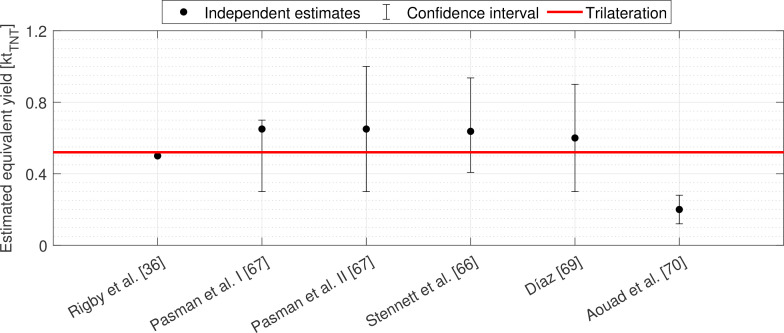
A comparison of trilateration's equivalent yield of the Beirut explosion with other inverse schemes.

Figure 11*a* shows TNT equivalent mass estimates in the range 0.5−0.525 kt_${,}_{\text{TNT}}$_ to occur most frequently, implying that the method can indeed identify the true yield, despite the presence of measurement noise, as the same value is consistently output when using different sensor combinations. This conclusion is reinforced by figure 11*b*, which shows that iterations in this bin exhibit the lowest mean internal error. Alignment of both metrics is needed for confidence in the solution since either can be skewed by noise in isolation. The iteration with minimum internal error over the 0.5−0.525 kt_${,}_{\text{TNT}}$_ range has an assumed yield of 0.52 kt_${,}_{\text{TNT}}$_. This falls well within the intervals determined by other researchers who use a range of established inverse methods to estimate the equivalent yield (but not the equivalent charge position) of the Beirut explosion [36,66,67,69,70], see figure 12.

**Figure 13.**
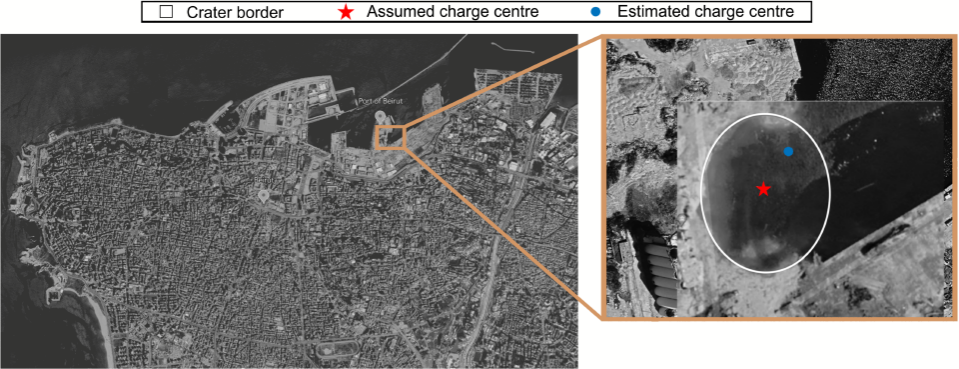
Contextualizing the accuracy of the equivalent charge position estimation. The crater border is as defined by Pasman *et al.* [67] and the satellite imagery is from Google Earth (March 2023).

The iteration with minimal internal error results in a radial error in the estimated equivalent charge position of 43.4 m (0.54 m/kg${{,}_{\text{TNT}}}^{1/3}$), assuming the centroid of the warehouse to be the true location. For scale, the estimated charge centre is within the 62 m radius crater produced by the blast [67], as shown in figure 13. Additionally, assuming the AN to have been formed into a perfect hemisphere with the density of loosely packed ANFO (0.8 g/cm^1/3^ [75]), it would have a radius of 11.8 m, meaning the charge position estimated by trilateration has an error of less than four charge radii from the assumed centre. Note, figure 11*c* shows substantial variability in the radial error of the position estimates, ranging from 1 to 200 m in the bin of interest, which results from noise in the dataset. It has been shown previously that position estimation can be improved substantially if the input data are screened for noise.

Overall, this section demonstrates the successful inverse analysis of the Beirut explosion by the trilateration algorithm. This is proof that the tool can function under in-field conditions of substantial noise with robustness and with accuracy comparable to other methods in terms of mass estimation, but with the added functionality of estimating charge centre. Additionally, for a given value of assumed charge mass, the average computational time for 10 000 iterations was just 3.9 s, highlighting the rapid nature of the trilateration scheme, even with its current ‘brute force’ exhaustive implementation.

## 6. Summary and conclusions

Post-blast decision-making and emergency response require an accurate and timely estimate of equivalent yield and charge centre. However, there currently exists no inverse blast analysis tool capable of achieving both of these concurrently, while also being robust against noise. This paper details the development and testing of a trilateration inverse scheme, based on the concept of GPS positioning.

In addition to being tested on artificial, idealized cases (with and without measurement noise), the inverse approach has seen successfully applied to two real-world scenarios using relative time of arrival data. First, in the well-controlled experimental work of Farrimond *et al.* [60], trilateration identified charge size with an error as low as 1%. Second, it output an equivalent mass for the Beirut explosion comparable with numerous established inverse methodologies [36,66,67,69], demonstrating its robustness to substantial measurement variability. Alongside the algorithm’s reliable and accurate assessment of charge mass, it established equivalent charge position estimations with errors as low as 0.1 m kg${{,}_{\text{TNT}}}^{1/3}$. Within the literature, there are very few inverse techniques capable of approximating charge centre at all [11,13,36,43,66,69]), but trilateration does so effectively. Considering that the centre of an explosion is a crucial starting point for other analyses, such as the prediction of the severity and distribution of structural damage and human injury, the concept developed in this paper has the potential to be a highly impactful and useful tool in high-speed inverse blast characterization.

## Data Availability

*rs_blast_trilateration.m* is provided as supplementary material [76].

## Footnotes

^1^
This polylogarithmic function was derived by Rigby *et al*. [36] as a simplified fit to the Kingery & Bulmash [32] arrival time relations.

^2^
A physical phenomenon wherein a larger yield explosion at a greater distance produces the same scaled effect as a small explosion nearby.

^3^
This is representative of threats on the scale of parcel bombs up to compact car bombs [37].

^4^
For reference, the total number of different 10 probe subsets from a set of 38 is given by the *n choose k* function, $C_{k}(n)$, with $C_{10}(38)=472\;733\;756$ unique combinations without repetition and irrespective of order.
